# Risk factors of early disease progression and decreased survival for multiple myeloma patients after upfront autologous stem cell transplantation

**DOI:** 10.1007/s00277-024-05641-y

**Published:** 2024-03-13

**Authors:** Te-Lin Hsu, Chun-Kuang Tsai, Chun-Yu Liu, Chiu-Mei Yeh, Fen-Lan Lin, Liang-Tsai Hsiao, Yao-Chung Liu, Sheng-Hsuan Chien, Hao-Yuan Wang, Po-Shen Ko, Ting-An Lin, Wen-Chun Chen, Po-Min Chen, Jin-Hwang Liu, Jyh-Pyng Gau, Chia-Jen Liu

**Affiliations:** 1 Division of Hematology, Department of Medicine, Taipei Veterans General Hospital, No. 201 Shipai Road, Sec. 2, Taipei, 11217 Taiwan; 2 Division of Holistic and Multidisciplinary Medicine, Department of Medicine, Taipei Veterans General Hospital, Taipei, Taiwan; 3 School of Medicine, National Yang Ming Chiao Tung University, Taipei, Taiwan; 4 Division of Transfusion Medicine, Department of Medicine, Taipei Veterans General Hospital, Taipei, Taiwan; 5 Institute of Public Health, National Yang Ming Chiao Tung University, Taipei, Taiwan; 6 Section of Hematology and Oncology, Department of Internal Medicine, Cheng Hsin General Hospital, Taipei, Taiwan; 7 Institute of Biopharmaceutical Sciences, National Yang Ming Chiao Tung University, Taipei, Taiwan; 8 Chong Hin Loon Memorial Cancer and Biotherapy Research Center, National Yang Ming Chiao Tung University, Taipei, Taiwan; 9 Institute of Emergency and Critical Care Medicine, National Yang Ming Chiao Tung University, Taipei, Taiwan

**Keywords:** Multiple myeloma, Autologous hematopoietic stem cell transplantation, Maintenance therapy, Monocyte count, Platelet count

## Abstract

Multiple myeloma (MM) stands as the second most prevalent hematological malignancy, constituting approximately 10% of all hematological malignancies. Current guidelines recommend upfront autologous stem cell transplantation (ASCT) for transplant-eligible MM patients. This study seeks to delineate factors influencing post–ASCT outcomes in MM patients. Our cohort comprised 150 MM patients from Taipei Veterans General Hospital, with progression-free survival (PFS) as the primary endpoint and overall survival (OS) as the secondary endpoint. A Cox proportional hazards model was employed to discern potential predictive factors for survival. ASCT age ≥ 65 (hazard ratio [HR] 1.94, 95% confidence interval [CI] 1.08–3.47) and the presence of extramedullary disease (HR 2.53, 95% CI 1.53–4.19) negatively impacted PFS. Conversely, treatment response ≥ VGPR before ASCT (HR 0.52, 95% CI 0.31–0.87) and total CD34^+^ cells collected ≥ 4 × 10^6^ cells/kg on the first stem cell harvesting (HR 0.52, 95% CI 0.32–0.87) were positively associated with PFS. For OS, patients with ISS stage III (HR 2.06, 95% CI 1.05–4.04), the presence of extramedullary disease (HR 3.92, 95% CI 2.03–7.58), light chain ratio ≥ 100 before ASCT (HR 7.08, 95% CI 1.45–34.59), post–ASCT cytomegalovirus infection (HR 9.43, 95% CI 3.09–28.84), and a lower conditioning melphalan dose (< 140 mg/m^2^; HR 2.75, 95% CI 1.23–6.17) experienced shorter OS. In contrast, post–ASCT day + 15 absolute monocyte counts (D15 AMC) > 500/µl (HR 0.36, 95% CI 0.17–0.79) and post–ASCT day + 15 platelet counts (D15 PLT) > 80,000/µl (HR 0.48, 95% CI 0.24–0.94) were correlated with improved OS. Significantly, early PLT and AMC recovery on day + 15 predicting longer OS represents a novel finding not previously reported. Other factors also align with previous studies. Our study provides real-world insights for post–ASCT outcome prediction beyond clinical trials.

## Introduction

Multiple myeloma (MM) is the second most common hematological malignancy and accounts for roughly 10% of all hematological malignancies [1]. The United States had an estimated lifetime incidence of MM at 0.76% in 2022, with 34,470 new cases reported annually [2]. Current treatment guidelines recommend upfront autologous stem cell transplantation (ASCT) for MM transplant-eligible patients, especially for those with high-risk cytogenetics [1, 3]. However, studies have shown that frontline ASCT primarily improves progression-free survival (PFS) rather than overall survival (OS) [4]. In the IFM 2009 trial [5], patients who received bortezomib, lenalidomide, and dexamethasone (VRD) followed by upfront ASCT demonstrated superior PFS (*p* < 0.001) compared to the non-ASCT group, but OS was comparable (*p* = 0.87). The recent DETERMINATION trial yielded similar results by comparing VRD alone with lenalidomide maintenance and VRD followed by ASCT with lenalidomide maintenance [6]. To date, several studies have addressed outcomes for MM patients who have undergone ASCT. However, the risk factors for poor outcomes vary between studies. Tandon et al. analyzed the outcomes of MM patients who received frontline treatment and found that patients with high-risk cytogenetics had poorer PFS and OS [7]. Factors such as response ≥ very good partial response (VGPR), creatinine ≥ 2 mg/dl and age were not significant. Recently, a large retrospective study reported that pre–ASCT treatment response and age can predict outcomes [8]. Another study evaluated the risk factors that impact post–ASCT outcomes for MM patients based on the Center for International Blood and Marrow Transplant Research (CIBMTR) database, finding that patients with treatment response ≥ VGPR, Karnofsky performance score ≥ 90, International Staging System (ISS) III compared to I–II, and high-risk cytogenetics were significant factors for post–ASCT PFS and OS prediction [9]. Moreover, post–ASCT measurable residual disease (MRD) has been shown to predict outcomes and guide post–transplant management in several studies [10–12]. However, routine MRD evaluation is limited due to financial constraints and availability. Thus, there is a need for affordable and easily accessible predictive factors for post–ASCT outcomes.

## Materials and methods

### Study population

This is a retrospective study. Patients at Taipei Veterans General Hospital newly diagnosed with MM according to International Myeloma Working Group (IMWG) criteria were enrolled in this study [13]. We screened MM patients diagnosed between October 1, 2006 and August 31, 2020. Patients who did not receive induction treatment followed by frontline ASCT were excluded. Institutional review board approval was obtained at Taipei Veterans General Hospital (no. 2022-05-001AC).

### Induction treatment, stem cell harvesting, and autologous stem cell transplantation

Induction treatment varies based on the timing of diagnosis and the availability of different treatment options. Chemotherapy-based regimens commonly include vincristine, doxorubicin, and dexamethasone (VAD), while novel agent-based regimens often use bortezomib, thalidomide, and dexamethasone (VTD).

All patients received stem cell mobilization with chemotherapy and granulocyte colony–stimulating factor (G-CSF) [14, 15]. Peripheral blood stem cell harvesting started when white cell count (WBC) was ≥ 1,000/µl and peripheral blood hematopoietic progenitor cells were ≥ 20 × 10^6^ cells/L [16, 17]. Leukapheresis was performed using the COBE Spectra apheresis system (Version 6.1, COBE Laboratories) with the processed volume of 10,000 ml or 2–3 times of total blood volume, depending on the patients’ body weight and age [18]. The collected CD34^+^ hematopoietic stem cell count was measured using flow cytometry. Before use, stem cells were preserved in liquid nitrogen freezers (MVE HEco 1500Series Freezer) [18]. The detailed apheresis procedure is outlined in our previous study [19].

Conditioning chemotherapy with melphalan was administered before ASCT [20]. Melphalan dosage depended on the patients’ age, Eastern Cooperative Oncology Group (ECOG) performance status, and the treating physician’s discretion [20]. On the day of ASCT, the cryopreserved stem cells were thawed in a thermostatic water bath (37 °C) and reinfused via a central venous catheter. A small number of thawed stem cells was sent for cell viability analysis and culture for quality control. G-CSF was prescribed after ASCT and until neutrophil engraftment. The treating physician decided on the starting day of G-CSF [21, 22].

### Data collection

Patient data were collected at multiple timepoints, including at MM diagnosis, before ASCT, on the day of ASCT, and after ASCT. Baseline information encompassed sex, ISS disease stage, ECOG performance status, disease characteristics, bone marrow pathology, comorbidities, induction treatment regimens, and laboratory data obtained at diagnosis. Extramedullary disease was recorded and defined as hematologenous spreading of MM according to an expert review conducted in 2021 [23]. Paraosseous plasmacytoma was not included in this definition. The total number of CD34^+^ cells collected on the first stem cell harvesting was also recorded. Before ASCT, defined as within one week before stem cell infusion, we collected conditioning melphalan dosage, light chain ratio, and treatment response according to IMWG response criteria [24]. On the day of ASCT, patients’ age at ASCT and the duration between stem cell mobilization and ASCT were collected. After ASCT, we collected laboratory data on day + 15 (D15), including absolute monocyte counts (AMC), absolute neutrophil counts (ANC), absolute lymphocyte counts (ALC), and platelet counts (PLT). Additionally, information on post–ASCT bortezomib maintenance, cytomegalovirus (CMV) infection within one year after ASCT, and fungal infection within one year after ASCT was recorded.

### Statistical analysis

Patients’ baseline information was categorized and presented as the total number (*n*) and proportion (%) for further analysis. The primary endpoint of this study was PFS. The secondary endpoint was OS. Patients lost to follow-up were censored. For patients who received ASCT followed by allogeneic hematopoietic stem cell transplantation (alloHSCT), the last follow-up date was considered as day 0 of alloHSCT. Hazard ratios (HRs) and 95% confidence intervals (CIs) were calculated using Cox proportional hazards models to analyze the impact of each factor on survival. A univariate Cox proportional hazards model identified potential risk factors for decreased survival, with those having *p* < 0.1 proceeding to backward multivariate Cox regression analysis. The cumulative incidence of disease progression and survival were calculated using the Kaplan–Meier method. The relationship between age ≥ 65 and melphalan dosage ≥ 180 mg/m^2^ was evaluated using a Chi-square test. SAS 9.4 (SAS Institute Inc., Cary, NC) and STATA 15.1 (StataCorp, College Station, TX) were used for data management and statistical analysis. Statistical significance was defined as *p* < 0.05.

## Results

### Clinical characteristics of the study population

**Fig. 1:**
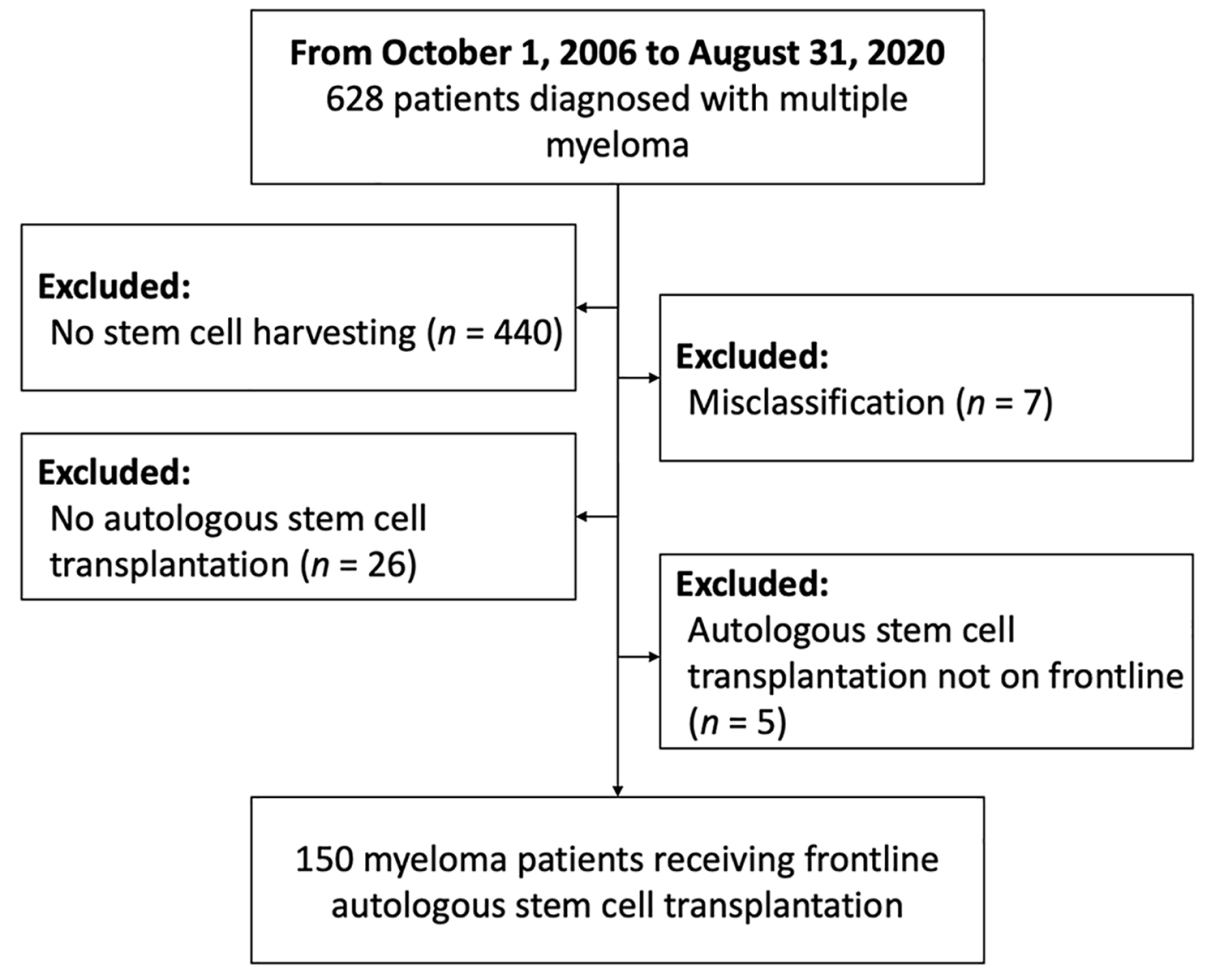
Patient selection flowchart

A total of 628 MM patients at Taipei Veterans General Hospital diagnosed between October 2006 and August 2020 were identified. After exclusions, the final study population consisted of 150 MM patients who underwent upfront ASCT (shown in Fig. 1). The median ASCT age of the patients was 58 (range, 24–75), with 34 patients (22.7%) aged ≥ 65 and 116 patients (77.3%) younger than 65 years. Among the study population, 54.7% were men. ECOG performance status at MM diagnosis with a score of 0–1 was reported in 115 patients (76.7%) and ≥ 2 in the remaining patients (23.3%). ISS stages I & II, and III were 74.7%, and 24.0%, respectively. Extramedullary disease was present in 25 cases (16.7%). Comorbidities including heart failure, chronic pulmonary disease, diabetes mellitus, and hypertension were reported in 2.0%, 4.0%, 7.3%, and 8.7% of the cases, respectively. Induction therapy consisted of VAD in 6.7% of the patients, VTD in 46.0%, bortezomib, cyclophosphamide, thalidomide, and dexamethasone (VCTD) in 14.0%, VCD in 10.7%, and other regimens in 22.7% of the patients. Treatment response ≥ VGPR before ASCT was identified in 125 patients (83.3%). A conditioning melphalan dosage of less than 140 mg/m^2^ was reported in 31 patients (20.7%), between 140 and 179 mg/m^2^ in 40 patients (26.7%), and ≥ 180 mg/m^2^ in the majority of the study population (79 cases, 57.2%). In the context of ASCT, conventional melphalan doses are 200 mg/m^2^, 140 mg/m^2^, and 100 mg/m^2^. Most of the conditioning dosages in our study deviated slightly from these standard targets due to various clinical situations (dosage adjustments for non-integer dosages, cost-effectiveness considerations, etc.). Furthermore, certain clinicians opted to reduce the intended conditioning dosage by 10–20%, considering the patient’s clinical condition, resulting in a dosage between 140 and 179 mg/m^2^. In patients aged ≥ 65, conditioning melphalan dosage ≥ 180 mg/m^2^ was 29.4%, and the percentage was significantly higher in the group aged < 65 (59.5%) (*p* = 0.002). Post–ASCT D15 laboratory data showed ANC > 2,000/µl in 66.7% of patients, ALC > 500/µl in 69.3%, AMC > 500/µl in 88.0%, and PLT > 80,000/µl in 49.3%. Bortezomib maintenance was given to 45.3% of patients, while CMV infection was reported in 4.7% and fungal infection in 2.0%. Detailed information on the patients’ characteristics is listed in Table 1.

**Table 1:** Baseline characteristics of myeloma patients receiving front-line autologous stem cell transplantation

Characteristics	Total *n* = 150 (%)
Median ASCT age, years (range)	58 (24–75)
< 65	116 (77.3)
≥ 65	34 (22.7)
Sex (male)	82 (54.7)
ECOG	
0–1	115 (76.7)
≥ 2	35 (23.3)
High-risk cytogenetics (conventional)	6 (4)
ISS stage	
I & II	112 (74.7)
III	36 (24.0)
Unknown	2 (1.3)
Heavy chain	
IgG	81 (54.0)
IgA	37 (24.7)
IgD	1 (0.7)
Light chain disease	31 (20.7)
Light chain	
Kappa	84 (56.0)
Lambda	66 (44.0)
Comorbidities	
Heart failure	3 (2.0)
Chronic pulmonary disease	6 (4.0)
Diabetes mellitus	11 (7.3)
Hypertension	13 (8.7)
Presence of extramedullary disease	25 (16.7)
Conditioning melphalan dose	
≥ 180 mg/m^2^	79 (52.7)
140–179 mg/m^2^	40 (26.7)
< 140 mg/m^2^	31 (20.7)
Duration between mobilization and ASCT ≥ 60 days	78 (52.0)
Light chain ratio ≥ 100 before ASCT	4 (2.7)
Treatment response ≥ VGPR before ASCT	125 (83.3)
Total CD34^+^ cells collected ≥ 4 × 10^6^ cells/kg on the first stem cell harvesting	114 (76.0)
Laboratory data on day + 15	
Absolute monocyte count > 500/µl	132 (88.0)
Absolute neutrophil count > 2,000/µl	100 (66.7)
Absolute lymphocyte count > 500/µl	104 (69.3)
Platelet > 80,000/µl	74 (49.3)
Post ASCT CMV infection	7 (4.7)
Post ASCT fungal infection	3 (2.0)
Laboratory data at MM diagnosis	
Plasma cells of bone marrow ≥ 60%	65/114 (57.0)
Hemoglobin < 10 g/dl	65/138 (47.1)
Platelet < 150,000/µl	30/137 (21.9)
Serum albumin < 3.5 g/dl	63/136 (46.3)
Corrected serum calcium ≥ 11 mg/dl	19/132 (14.4)
Serum creatinine ≥ 2 mg/dl	19/139 (13.7)
Lactate dehydrogenase ≥ 250 U/L	24/137 (17.5)
First-line therapy	
VAD	10 (6.7)
VTD	69 (46.0)
VCTD	21 (14.0)
VCD	16 (10.7)
Others	34 (22.7)
Post ASCT bortezomib maintenance	68 (45.3)

ASCT, autologous stem cell transplantation; ECOG, Eastern Cooperative Oncology Group performance; ISS, International Staging System; VGPR, very good partial response; CMV, cytomegalovirus; VAD, vincristine, doxorubicin, and dexamethasone; VTD, bortezomib, thalidomide, and dexamethasone; VCTD, bortezomib, cyclophosphamide, thalidomide, and dexamethasone VCD, bortezomib, cyclophosphamide, dexamethasone

### Risk factors for disease progression after autologous stem cell transplantation

**Fig. 2:**
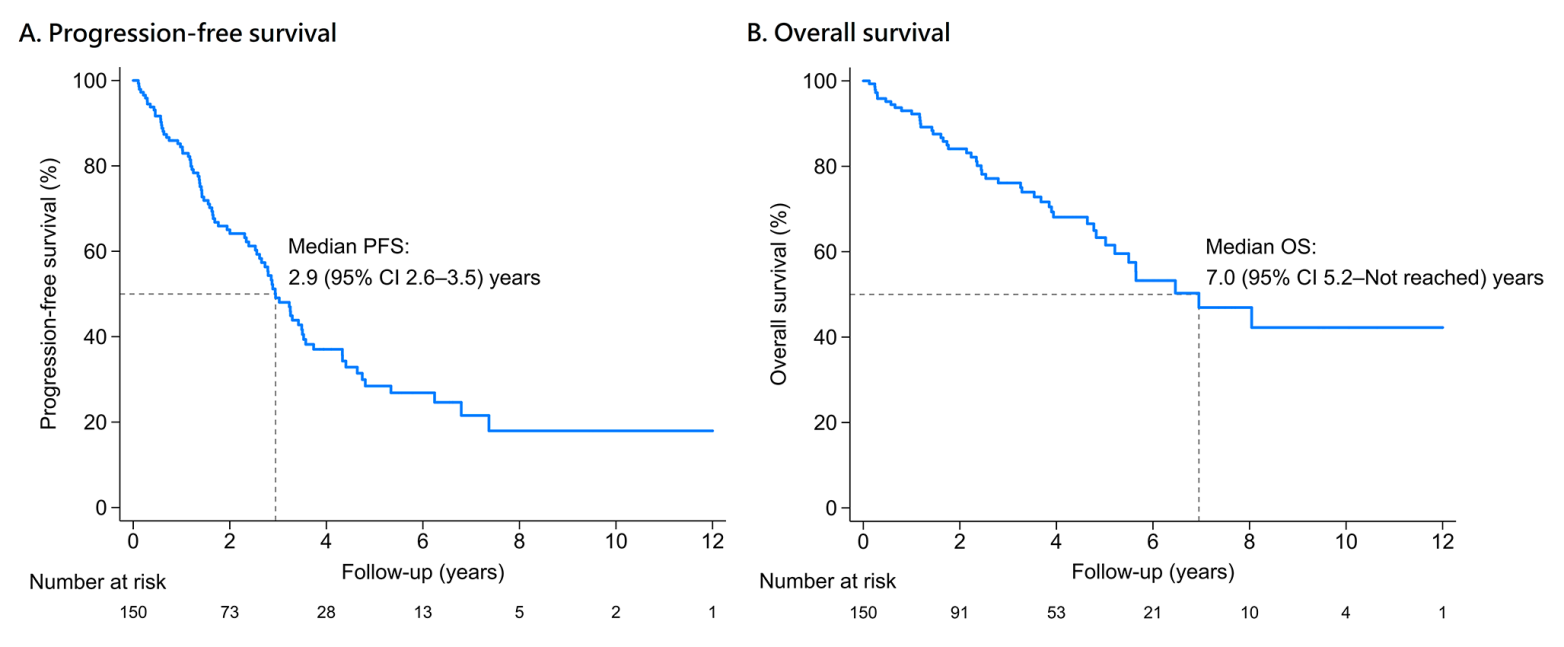
Kaplan–Meier survival curves of progression-free survival and overall survival

The median PFS was 2.9 years, with the cumulative incidence shown in Fig. 2. Among the variables, those with a *p* value of < 0.1 in the univariate analysis were identified as possible risk factors associated with PFS and underwent multivariate analysis (Table 2). In the backward multivariate analysis, ASCT age ≥ 65 (HR 1.94, 95% CI 1.08–3.47) and the presence of extramedullary disease (HR 2.53, 95% CI 1.53–4.19) were factors negatively associated with PFS. On the other hand, treatment response ≥ VGPR before ASCT (HR 0.52, 95% CI 0.31–0.87) and total CD34^+^ cells collected ≥ 4 × 10^6^ cells/kg on the first stem cell harvesting (HR 0.52, 95% CI 0.32–0.87) were significant predictors positively associated with PFS after ASCT (Table 2).

**Table 2:** Risk factors for disease progression of myeloma patients receiving front-line autologous stem cell transplantation

Predictive variables	Univariate analysis		Multivariate analysis^a^	
	HR (95% CI)	*P* value	HR (95% CI)	*P* value
Median ASCT age, years (range)				
< 65	reference			
≥ 65	2.20 (1.25–3.89)	0.006	1.94 (1.08–3.47)	0.027
Sex (male)	1.16 (0.75–1.80)	0.499		
ECOG				
0–1	reference			
≥ 2	1.33 (0.83–2.13)	0.231		
ISS stage				
I & II	reference			
III	1.33 (0.82–2.18)	0.252		
Unknown	-			
Comorbidities				
Heart failure	1.30 (0.32–5.33)	0.711		
Chronic pulmonary disease	0.23 (0.03–1.67)	0.147		
Diabetes mellitus	1.32 (0.61–2.87)	0.486		
Hypertension	1.53 (0.70–3.35)	0.285		
Presence of extramedullary disease	2.62 (1.60–4.29)	< 0.001	2.53 (1.53–4.19)	< 0.001
Conditioning melphalan dose				
≥ 180 mg/m^2^	reference			
140–179 mg/m^2^	1.23 (0.74–2.14)	0.388		
< 140 mg/m^2^	1.70 (0.99–2.94)	0.056		
Duration between mobilization and ASCT ≥ 60 days	1.10 (0.71–1.70)	0.666		
Light chain ratio ≥ 100 before ASCT	1.68 (0.53–5.32)	0.381		
Treatment response ≥ VGPR before ASCT	0.49 (0.30–0.80)	0.005	0.52 (0.31–0.87)	0.012
Total CD34^+^ cells collected ≥ 4 × 10^6^ cells/kg on the first stem cell harvesting	0.54 (0.33–0.87)	0.012	0.52 (0.32–0.87)	0.012
Laboratory data on day + 15				
Absolute monocyte count > 500/µl	0.55 (0.29–1.04)	0.067		
Absolute neutrophil count > 2,000/µl	0.65 (0.42–1.01)	0.056		
Absolute lymphocyte count > 500/µl	1.19 (0.74–1.92)	0.463		
Platelet > 80,000/µl	0.66 (0.43–1.02)	0.062	0.67 (0.42–1.07)	0.091
Post ASCT CMV infection	1.99 (0.87–4.59)	0.105		
Post ASCT fungal infection	1.25 (0.40–3.98)	0.701		
Laboratory data at MM diagnosis				
Plasma cells of bone marrow ≥ 60%	.			
Hemoglobin < 10 g/dl	1.49 (0.95–2.31)	0.081		
Platelet < 150,000/µl	1.24 (0.73–2.11)	0.427		
Serum albumin < 3.5 g/dl	1.32 (0.85–2.06)	0.223		
Corrected serum calcium ≥ 11 mg/dl	1.47 (0.82–2.63)	0.191		
Serum creatinine ≥ 2 mg/dl	0.92 (0.47–1.78)	0.802		
Lactate dehydrogenase ≥ 250 U/L	1.19 (0.67–2.12)	0.554		
First-line therapy				
VAD	reference			
VTD	0.34 (0.16–0.72)	0.005		
VCTD	0.36 (0.15–0.87)	0.023		
VCD	0.56 (0.24–1.36)	0.2		
Others	0.48 (0.22–1.03)	0.06		
Post ASCT bortezomib maintenance^b^	0.62 (0.39–0.97)	0.036		

HR, Hazard ratio; CI, confidence interval; ASCT, autologous stem cell transplantation; ECOG, Eastern Cooperative Oncology Group performance; ISS, International Staging System; VGPR, very good partial response; CMV, cytomegalovirus; VAD, vincristine, doxorubicin, and dexamethasone; VTD, bortezomib, thalidomide, and dexamethasone; VCTD, bortezomib, cyclophosphamide, thalidomide, and dexamethasone VCD, bortezomib, cyclophosphamide, dexamethasone

^a^All factors with *p* < 0.1 in the univariate analysis were selected for the backward multivariate Cox regression model

^b^ Treatment was analyzed as a time-dependent covariate in the Cox regression model

### Risk factors for decreased survival after autologous stem cell transplantation

Variables with a *p* < 0.1 in the univariate analysis were selected for the multivariate analysis (Table 3). In the backward multivariate Cox regression model, ISS stage III, compared to stage I & II (HR 2.06, 95% CI 1.05–4.04), the presence of extramedullary disease (HR 3.92, 95% CI 2.03–7.58), light chain ratio ≥ 100 before ASCT (HR 7.08, 95% CI 1.45–34.59), post–ASCT CMV infection (HR 9.43, 95% CI 3.09–28.84), and a lower conditioning melphalan dose (< 140 mg/m^2^; HR 2.75, 95% CI 1.23–6.17) were factors associated with a shorter OS. In contrast, D15 AMC > 500/µl (HR 0.36, 95% CI 0.17–0.79) and D15 PLT > 80,000/µl (HR 0.48, 95% CI 0.24–0.94) were significant factors positively associated with overall survival (Table 3). The Kaplan–Meier estimator depicted the cumulative probability of the death curve (Fig. 2), with a median overall survival of 7.0 years.

**Table 3:** Risk factors for decreased survival of myeloma patients receiving front-line autologous stem cell transplantation

Predictive variables	Univariate analysis		Multivariate analysis^a^	
	HR (95% CI)	*P* value	HR (95% CI)	*P* value
Median ASCT age, years (range)				
< 65	reference			
≥ 65	2.37 (1.12–5.01)	0.024		
Sex (male)	1.24 (0.69–2.21)	0.473		
ECOG				
0–1	reference			
≥ 2	1.47 (0.80–2.69)	0.217		
ISS stage				
I & II	reference			
III	2.26 (1.25–4.09)	0.007	2.06 (1.05–4.04)	0.035
Unknown	-			
Comorbidities				
Heart failure	2.82 (0.67–11.85)	0.157		
Chronic pulmonary disease	0.00 (0.00–.)	0.986		
Diabetes mellitus	2.55 (1.14–5.72)	0.023		
Hypertension	2.37 (0.91–6.12)	0.076		
Presence of extramedullary disease	3.51 (1.94–6.33)	< 0.001	3.92 (2.03–7.58)	< 0.001
Conditioning melphalan dose				
≥ 180 mg/m^2^	reference			
140–179 mg/m^2^	1.12 (0.53–2.37)	0.760	1.82 (0.79–4.21)	0.162
< 140 mg/m^2^	1.96 (0.97–3.97)	0.061	2.75 (1.23–6.17)	0.014
Duration between mobilization and ASCT ≥ 60 days	1.67 (0.92–3.04)	0.091		
Light chain ratio ≥ 100 before ASCT	4.30 (1.32–14.05)	0.016	7.08 (1.45–34.59)	0.016
Treatment response ≥ VGPR before ASCT	0.49 (0.26–0.93)	0.029		
Total CD34^+^ cells collected ≥ 4 × 10^6^ cells/kg on the first stem cell harvesting	0.71 (0.37–1.38)	0.310		
Laboratory data on day + 15				
Absolute monocyte count > 500/µl	0.31 (0.15–0.62)	0.001	0.36 (0.17–0.79)	0.010
Absolute neutrophil count > 2,000/µl	0.90 (0.50–1.64)	0.731		
Absolute lymphocyte count > 500/µl	0.80 (0.43–1.46)	0.459		
Platelet > 80,000/µl	0.43 (0.24–0.79)	0.006	0.48 (0.24–0.94)	0.032
Post ASCT CMV infection	5.50 (2.28–13.27)	< 0.001	9.43 (3.09–28.84)	< 0.001
Post ASCT fungal infection	3.55 (1.09–11.60)	0.036		
Laboratory data at MM diagnosis				
Plasma cells of bone marrow ≥ 60%	.			
Hemoglobin < 10 g/dl	1.80 (0.97–3.35)	0.061		
Platelet < 150,000/µl	1.56 (0.80–3.04)	0.197		
Serum albumin < 3.5 g/dl	1.33 (0.73–2.43)	0.354		
Corrected serum calcium ≥ 11 mg/dl	1.00 (0.45–2.26)	0.993		
Serum creatinine ≥ 2 mg/dl	1.31 (0.58–2.93)	0.516		
Lactate dehydrogenase ≥ 250 U/L	1.86 (0.92–3.79)	0.087		
First-line therapy				
VAD	reference			
VTD	0.22 (0.09–0.53)	0.001		
VCTD	0.19 (0.06–0.59)	0.004		
VCD	0.30 (0.10–0.87)	0.027		
Others	0.36 (0.15–0.86)	0.021		
Post ASCT bortezomib maintenance^b^	0.46 (0.24–0.89)	0.021		

HR, Hazard ratio; CI, confidence interval; ASCT, autologous stem cell transplantation; ECOG, Eastern Cooperative Oncology Group performance; ISS, International Staging System; VGPR, very good partial response; CMV, cytomegalovirus; VAD, vincristine, doxorubicin, and dexamethasone; VTD, bortezomib, thalidomide, and dexamethasone; VCTD, bortezomib, cyclophosphamide, thalidomide, and dexamethasone VCD, bortezomib, cyclophosphamide, dexamethasone

^a^All factors with *p* < 0.1 in the univariate analysis were selected for the backward multivariate Cox regression model

^b^ Treatment was analyzed as a time-dependent covariate in the Cox regression model

## Discussion

In this study, we observed that age ≥ 65 at ASCT and the presence of extramedullary disease were associated with shorter PFS. Conversely, treatment response ≥ VGPR before ASCT and total CD34^+^ cells collected ≥ 4 × 10^6^ cells/kg on the first stem cell harvesting correlated with prolonged time to disease progression. Factors impacting survival were further analyzed, revealing that extramedullary disease, light chain ratio ≥ 100 before ASCT, post–ASCT CMV infection, a lower conditioning melphalan dose (< 140 mg/m^2^ compared with ≥ 180 mg/m^2^), and ISS stage III at MM diagnosis were linked to shorter survival. In contrast, D15 AMC > 500/µl and D15 PLT > 80,000/µl were associated with extended survival. Notably, D15 AMC and PLT were identified as predictors of improved OS for MM patients undergoing ASCT, which is a novel finding. Additionally, extramedullary disease corresponded to shorter PFS and OS.

To the best of our knowledge, this is the first study describing D15 PLT as a predictive factor for survival. Some studies have reported that post–ASCT PLT recovery can affect outcomes, but not as early as our finding. We also identified a trend toward better PFS in patients with D15 PLT > 80,000/µl. Ninan et al. found that post–ASCT PLT ≥ 150,000/µl was associated with better OS (*p* < 0.001), with a shorter time to reach PLT > 50,000/µl in this group (17.2 days vs. 26.5 days, *p* < 0.001) [25]. This result reveals that early PLT recovery predicts better OS in patients who have undergone ASCT. A recent nationwide Japanese study evaluated the impact of the delay of platelet recovery after ASCT, defined as day + 28 PLT < 50,000/µl or PLT transfusion dependence, on survival in non-Hodgkin lymphoma and MM patients. They found that both lymphoma and MM patients with delayed PLT recovery had worse OS and PFS. Furthermore, they reported that, in addition to total CD34^+^ cells infused, patient factors including disease status at ASCT and ISS stage can affect PLT recovery, implying that the bone marrow microenvironment may have a role in PLT recovery. Previous studies have shown that impaired bone marrow microenvironment can impact PLT recovery and bone marrow function [26–28]. It has also been reported that megakaryocytes have a supportive role in expanding hematopoietic stem cells, confirmed in murine models [29, 30]. Collectively, the predictive value of D15 PLT > 80,000/µl may reflect a better bone marrow function, which is correlated with an improved OS. Similarly, several studies have shown that CD34^+^ yielded at a dose of 4 × 10^6^ cells/kg or higher after stem cell mobilization improves post–ASCT survival in MM patients, probably due to the relatively healthy bone marrow stem cell niches and microenvironment [31–34].

Early monocyte recovery is another novel finding in our work. Although there is some evidence addressing the early recovery of monocytes conferring better survival, no studies have reported results as early as ours. Turcotte et al. reported that early recovery of monocytes on day + 28 in patients undergoing hematopoietic stem cell transplantation is linked to longer survival [35]. Similarly, Dhakal et al. found that MM patients who received alloHSCT and had AMC > 300/µl at day + 60 and + 100 exhibited improved OS [36]. DeCook et al. observed that AMC > 300/µl at day + 100 after alloHSCT led to improved OS in myeloid and lymphoid malignancies [37]. These studies reveal that early AMC recovery is correlated with prolonged survival, and the benefits of early AMC recovery can be applied to different types of hematological malignancies after transplantation. Binder et al. used AMC and ALC as biomarkers of immune recovery in MM patients [38]. Immune reconstitution at post–MM treatment one month, defined as the recovery of AMC and ALC to normal reference range, correlated with significantly longer survival (HR 0.63, 95% CI 0.50–0.80, *p* < 0.001). Most patients with immune dysregulation at diagnosis had monocytopenia and increased to the normal range (> 500/µl) after immune recovery. In brief, early AMC recovery may reflect early immune reconstruction and better bone marrow function, thus prolonging survival [39].

Our data suggests poorer PFS and significantly worse OS in the lower-dose melphalan group, corroborating previous studies showing improved outcomes with higher melphalan doses [9, 40]. Despite age-related effects observed in the univariate analysis, they weren’t considered in the multivariate analysis due to model selection limitations imposed by the small sample size.

The presence of extramedullary disease significantly diminished both PFS and OS in this study, aligning with findings from prior investigations [41, 42]. We can also confirm that advanced ISS stage was predictive of poor outcomes after ASCT [43–48]. Additionally, MM patients with high light chain ratio may have more aggressive disease and a lower VGPR rate [49]. Several studies have also reported the correlation between treatment response before ASCT and post–ASCT outcomes [50–52]. These results are similar to those in our study. Another study conducted by Brioli et al. aimed to evaluate the relationship between treatment response before ASCT and patient outcomes [53]. They found that a response of ≥ VGPR before ASCT was the strongest predictor for better OS and PFS. However, following ASCT, VGPR failed to predict PFS in high-risk and ultra-high-risk patients identified through fluorescence in situ hybridization (FISH). Even after induction, VGPR did not correlate with improved PFS in ultra-high-risk patients. These observations lead to the conclusion that treatment response and cytogenetic risk features exert independent influences on outcomes. Additionally, it was noted that a favorable treatment response does not necessarily translate into better outcomes for high-risk and ultra-high-risk patients.

Lenalidomide is the only category 1 recommended post–ASCT maintenance therapy for MM, supported by clinical trials [54, 55]. In our study, bortezomib demonstrated significant PFS and OS improvement in the univariate analysis, aligning with documented efficacy in studies, although it was not selected for multivariate analysis, probably due to the small sample size [56–58]. These findings suggest that bortezomib can be a viable option for maintenance therapy in MM patients after ASCT, expanding the available choices beyond lenalidomide.

Our findings reveal a higher risk of early death associated with post–ASCT CMV infection. Despite guidelines recommending selective CMV monitoring, reactivation is not uncommon after ASCT [59–63]. In our institution, CMV monitoring focuses on critical cases. Our results imply that CMV infection should be kept in mind after ASCT, although regular monitoring may be unnecessary.

This retrospective study has limitations. Variations in induction therapy, conditioning melphalan dosage, and post–ASCT maintenance therapy were observed. The considerable variability in treatment regimens stems from the extended duration of treatment in our study group and the ongoing evolution of novel agents. Various induction regimens may potentially influence our results. Additionally, the FISH-based high-risk cytogenetic testing was limited because the test was not covered by Taiwan National Health Insurance. However, these limitations reflect real-world MM management and diverse patient populations. The main maintenance regimen was bortezomib due to coverage restrictions on lenalidomide by Taiwan National Health Insurance, which may affect risk factors. Only 10% of our patients received lenalidomide maintenance. Nonetheless, our data suggests that bortezomib can be a candidate of viable maintenance therapy options. The prevalence of D15 AMC > 500/µl in 88% of patients restricts its utility as a prognostic marker for reduced survival in MM, as it would only identify a minority (12%) percentage of patients with an unfavorable prognosis. Lastly, our sample size was small, possibly reducing statistical power and masking certain prognostic factors. Some of our factors were significant in the univariate analysis but were not selected for multivariate analysis by model selection. Larger studies are needed to address this issue.

## Conclusion

We discovered previously unreported predictors of better OS after ASCT in MM patients: post–ASCT D15 AMC > 500/µl and PLT > 80,000/µl. Additionally, our findings demonstrate the negative impact of extramedullary disease on outcomes. Other factors influencing post–ASCT outcomes in MM patients include age ≥ 65, treatment response ≥ VGPR before ASCT, light chain ratio ≥ 100 before ASCT, post–ASCT CMV infection, ISS stage III at MM diagnosis, and higher conditioning melphalan dosage ≥ 180 mg/m^2^. This real-world data provides valuable information for outcome prediction beyond clinical trials.

## Data Availability

The data of this study are not publicly available because the information can compromise the privacy of research participants, and the participants do not consent to public data release. Reasonable data requests can be directed to the corresponding author.
